# Ethnicity and survival in bladder cancer: a population-based study based on the SEER database

**DOI:** 10.1186/s12967-020-02308-w

**Published:** 2020-03-30

**Authors:** Wei Fang, Zhi-Yan Yang, Ting-Yu Chen, Xian-Feng Shen, Chao Zhang

**Affiliations:** 1 Center for Evidence-Based Medicine and Clinical Research, Taihe Hospital, Hubei University of Medicine, No. 32, South Renmin Road, Shiyan, 442000 China; 2 Department of General Surgery, Taihe Hospital, Hubei University of Medicine, Shiyan, 442000 China

**Keywords:** Bladder cancer, Ethnic, Survival rate, Kaplan–Meier survival, SEER database

## Abstract

**Background:**

Bladder cancer is the most common cancer in the urinary system and the fourth most common cancer in males. This study aimed to examine differences in the survival of bladder cancer patients of different ethnicities.

**Method:**

We used the SEER database to obtain data pertaining to bladder cancer patients from 2010 to 2015. Univariate and multivariate Cox proportional hazards regression analyses were used to estimate hazard ratios (HRs) and 95% confidence intervals (CIs) for the association between ethnicity and death. Kaplan–Meier survival and nomogram analyses were used to compare survival differences among patients with different ethnicities.

**Results:**

Among 101,364 bladder cancer patients, 90,910 were white, 5893 were black, 337 were American Indian/Alaska Native (AIAN), and 4224 were Asian or Pacific Islander (API). Our multivariate analysis identified differences between different ethnicities. Compared to the API group, the AIAN (HR = 1.31, 95% CI = 1.09–1.57, P < 0.001), black (HR = 1.56, 95% CI = 1.46–1.67, P < 0.001), and white (HR = 1.18, 95% CI = 1.12–1.25, P < 0.001) groups showed lower survival probabilities. Based on data from all Kaplan–Meier survival curves, there was no significant difference in survival between the black and AIAN groups, but the survival of these two races was worse than that of the white and API groups. We also used a nomogram to estimate patient survival and validated its predictive value.

**Conclusion:**

Our results suggest that ethnic differences exist in patients with bladder cancer, that the survival of black and AIAN bladder cancer patients is worse than that of other ethnicities and that the survival of API patients is the best. The significant prognostic factors of overall survival, which include age, sex, ethnicity, summary stage, American Joint Committee on Cancer stage, surgery type, and histologic type, should be applied to bladder cancer patient prognostication.

## Background

Bladder cancer is the ninth most common cancer in the world and the thirteenth leading cause of cancer-related death [1]. Among men, bladder cancer is the fourth most common cancer [2]. Bladder cancer is one of the most common cancers of the urinary system, and 80,470 new bladder cancer cases and 17,670 related deaths are expected to occur in the United States in 2019 [3]. Bladder cancer is generally divided into the following types: transitional cell carcinoma, squamous cell carcinoma, adenocarcinoma, small cell carcinoma, and sarcoma [4]. The most common type is transitional cell carcinoma, which is commonly referred to as urothelial carcinoma [5] because in many cases, bladder cancer begins in the urothelial cells that lie inside the bladder. The clinical symptoms of bladder cancer include hematuria, painful urination, pelvic pain, back pain, and frequent urination [6]. By 2020, the cost of bladder cancer treatment will reach $4.65 billion, which will impose a heavy burden on the social economy as well as on medical care [7]. Currently, the most widespread treatments used in clinical practice are surgery, chemotherapy in the bladder (intravesical chemotherapy), chemotherapy for the whole body (systemic chemotherapy), reconstruction, radiation therapy, and immunotherapy [8].

Previous studies have shown that sex, age, smoking, arsenic in drinking water, and ethnicity can affect the risk of bladder cancer [9–14]. Some studies have shown that the number of male patients affected by bladder cancer is three to four times that of female patients [15]. The risk of bladder cancer increases with age: more than 90% of patients are over 55 years old, and the average age is approximately 73 years old [3]. People who smoke are also more likely to have bladder cancer than those who do not smoke [16, 17]. The presence of arsenic in drinking water dramatically increases the risk of bladder cancer [18]. White individuals are also more likely to develop bladder cancer than individuals of other ethnicities. Although previous literature has reported racial differences in bladder cancer, these studies only show racial differences in bladder cancer incidence, and studies on bladder cancer survival in different ethnicities are very limited. The survival probability is a prediction of the likelihood that a patient will continue to survive. Because each survival probability is a prediction based on certain conditions, and we cannot consider all the conditions, the survival probability can only be used as a reference, and the survival possibility cannot provide a positive answer.

The aim of this study was to compare bladder cancer survival among different ethnicities in the United States. The data for this study were obtained from the National Cancer Institute’s Surveillance, Epidemiology, and End Results (SEER) database. Kaplan–Meier analysis was used to compare the survival of bladder cancer patients in different groups. Determining differences in bladder cancer survival between different ethnicities will improve the ability to predict the survival of patients in clinical practice.

## Methods

### Data source

The data for this study are from the National Cancer Institute’s SEER database. SEER*Stat version 8.3.5 (http://seer.cancer.gov/seerstat/) was used to extract data, select cases, and define variables. The case listing was based on the dataset of incidence-SEER 18 Registers Research Data + Hurricane Katrina Impacted Louisiana Cases, Nov 2018 Sub (1973-2016 varying).

### Population selection and classification

In this study, we chose patients with a diagnosis of malignant bladder cancer by positive histology who were diagnosed between 2010 and 2015. Patients were excluded if the diagnosis of bladder cancer was made at autopsy or was found in the death certificate. The exclusion criteria in our study were as follows: (a) unknown ethnicity; (b) unknown survival time; (c) unknown AJCC stage; (d) unknown summary stage; and (e) unknown surgical status.

We divided patient age into five subgroups: 0–50 years old, 51–60 years old, 61–70 years old, 71–80 years old, and 81+ years old. Marital status was also divided into five subgroups: single, married, separated/divorced, widowed, and unknown. Based on the ICD-O-3, we divided the histological type into epithelial cell carcinoma, squamous cell carcinoma, transitional cell carcinoma, adenocarcinoma, and unspecified carcinoma.

### Definition of stage

This study used two tumor grading methods: Summary Stage 2000 (1988+) and derived American Joint Committee on Cancer (AJCC) Stage Group, 7th ED (2010–2015). The Summary Stage is a simplified version of the stage, where the grade options are in situ, localized, regional, distant and unknown. It is also used in the SEER cancer Statistics Review and more recent SEER publications [19]. In contrast, the AJCC “Stage Group” [20] component is derived from collaborative stage coded fields, uses the collaborative stage algorithm and is effective in diagnoses given between 2010 and 2015 [21]. The AJCC “Stage Group” method is more elaborate than the Summary Stage 2000 method. Although the AJCC grading method is relatively new, it is more suitable for future studies and is more detailed than the Summary Stage method. As a result, our research was mainly based on the “Stage Group” method.

### Statistical analysis

A Chi square test was used to analyze factors related to ethnicity. When the P value was less than 0.01, it indicated that the ethnic group contributed to differences between groups. Univariate and multivariate hazard analyses were conducted using the Cox proportional hazards model to identify independent prognostic factors, and significance was set at a probability value of less than 0.05. First, we conducted univariate analysis, and then variables with a P value less than 0.05 were included in multivariate analysis. We used hazard ratios (HRs) with 95% confidence interval (CIs) to compare the survival risks of each population. When the value of the HR is greater than 1.0, it indicates that the degree of danger is increased compared with that in the reference group. When the value of HR is less than 1.0, it indicates that the degree of danger is decreased compared to that in the reference group. When the value of HR is equal to 1.0, it indicates that there is no effect [22]. Kaplan–Meier survival analysis was used to assess and compare the disease-related survival of patients with various variables. We generated survival curves based on this analysis and used the log-rank test to compare the significance of the curves. All the above methods were performed in R (Version 3.6.0; R Foundation).

### Nomogram construction

We used R 3.1.1 to build a nomogram that was based on the results of multivariate analysis of the Cox proportional hazards model. The maximum score for each variable was set to 10. The prognostic nomogram included all significant independent factors for overall survival (OS) in bladder cancer patients.

## Result

### Baseline characteristics

A total of 101,364 eligible bladder cancer patients from 2010 to 2015 were included in our study cohort through the SEER database. Table 1 shows the basic characteristics of the patients and the Chi square test results for the comparison of bladder cancer patients with different ethnicities. Among all patients, 90,910 (89.52%) patients were white, 5893 (5.80%) were black, 337 (0.33%) were American Indian/Alaska Native (AIAN), and 4224 (4.36%) were Asian or Pacific Islander (API). According to the results of the Chi square test, age at diagnosis (P < 0.001), sex (P < 0.001), summary stage (P < 0.001), AJCC stage (P < 0.001), marital status (P < 0.001), surgery (P < 0.001) and histologic type (P < 0.001) were all factors that were significantly different among ethnicities.

**Table 1:** Baseline characteristics of different ethnicities and all whole cohort

Factors	White		Black		AIAN		API		P-value	All ethnicities	
	Count	%	Count	%	Count	%	Count	%		Count	%
Total	90,910	89.51	5893	5.80	337	0.33	4424	4.36		101,364	100
Gender											
Female	20,830	22.91	1869	31.72	90	26.71	1080	25.57	< 0.001	23,869	23.55
Male	70,080	77.09	4024	68.28	247	73.29	3144	74.43		77,495	76.45
Age (years)											
00–50	3823	4.21	373	6.33	26	7.72	189	4.47	< 0.001	4411	4.35
51–60	11,601	12.76	1085	18.41	68	20.18	552	13.07		13,306	13.13
61–70	24,607	27.07	1738	29.49	101	29.97	1071	25.36		27,517	27.15
71–80	27,765	30.54	1613	27.37	86	25.52	1292	30.59		30,756	30.34
81+	23,114	25.43	1084	18.39	56	16.62	1120	26.52		25,374	25.03
Summary stage											
In situ	47,646	52.41	2502	42.46	147	43.62	2083	49.31	< 0.001	52,378	51.67
Localized	33,004	36.30	2322	39.40	122	36.20	1615	38.23		37,063	36.56
Regional	6346	6.98	598	10.15	36	10.68	337	7.98		7317	7.22
Distant	3,914	4.31	471	7.99	32	9.50	189	4.47		4606	4.54
Marital											
Single	9369	10.31	1375	23.33	45	13.35	378	8.95	< 0.001	11,167	11.02
Married	53,735	59.11	2416	41.00	177	52.52	2806	66.43		59,134	58.34
Separated/divorced	7844	8.63	780	13.24	38	11.28	238	5.63		8900	8.78
Widowed	12,711	13.98	888	15.07	41	12.17	522	12.36		14,162	13.97
Unknown	7251	7.98	434	7.36	36	10.68	280	6.63		8001	7.89
Surgery											
Yes	86,113	94.72	5494	93.23	313	92.88	4033	95.48	< 0.001	95,953	94.66
No	4797	5.28	399	6.77	24	7.12	191	4.52		5411	5.34
AJCC stage											
Stage 0a	44,309	48.74	2333	39.59	138	40.95	1944	46.02	< 0.001	48,724	48.07
Stage 0is	4316	4.75	229	3.89	14	4.15	172	4.07		4731	4.67
Stage I	21,314	23.45	1439	24.42	61	18.10	1088	25.76		23,902	23.58
Stage II	11,162	12.28	859	14.58	58	17.21	514	12.17		12,593	12.42
Stage III	3521	3.87	344	5.84	19	5.64	202	4.78		4086	4.03
Stage IV	6288	6.92	689	11.69	47	13.95	304	7.20		7328	7.23
Histologic type											
Epithelial	1399	1.54	126	2.14	8	2.37	58	1.37	< 0.001	1593	1.57
Squamous	1270	1.40	164	2.78	10	2.97	52	1.23		1498	1.48
Transitional	87,137	95.85	5419	91.96	301	89.32	4039	95.62		96,950	95.65
Adenocarcinomas	652	0.72	101	1.71	11	3.26	55	1.30		821	0.81
Unspecified	452	0.50	83	1.41	7	2.08	20	0.47		562	0.55

*AIAN* American Indian/Alaska Native, *API* Asian or Pacific Islander

Men comprised the majority of patients in each group, particularly in whites, where males accounted for 77.09%. In each group, patients aged 61+ years accounted for 71–83% of the group, and married was the most common marital status. Regardless of ethnicity, the proportion of patients who were treated with surgery exceeded 90%. Based on the summary stage and AJCC stage, most patients were in the early stages of cancer (in situ, localized, stage 0a, stage 0is, stage I, and stage II). Based on the histologic type, the highest proportion of patients likely had transitional cell carcinoma.

### Cox proportional hazards model

The results of the univariate and multivariate analyses are shown in Table 2. Univariate analysis showed that age (P < 0.001), sex (P < 0.001), ethnicity (P < 0.001), summary stage (P < 0.001), AJCC stage (P < 0.001), surgery (P < 0.001) and histologic type (P < 0.001) were significant prognostic factors of OS. The variables in univariate analysis with a P value of less than 0.05 were applied to multivariate analysis. We compared risks by comparing the HRs of the multivariate analysis results.

**Table 2:** Cox proportional-danger model analysis of bladder cancer patients

Factors	Univariate analysis		Multivariable analysis	
	HR (95% CI)	P	HR (95% CI)	P
Gender		< 0.001		
Male	Reference		Reference	
Female	1.10 (1.10–1.10)	< 0.001	0.99 (0.96–1.01)	0.336
Age (years)		< 0.001		
00–50	Reference		Reference	
51–60	1.30 (1.20–1.50)	< 0.001	1.34 (1.23–1.46)	< 0.001
61–70	1.60 (1.50–1.80)	< 0.001	1.70 (1.57–1.85)	< 0.001
71–80	2.60 (2.40–2.80)	< 0.001	2.80 (2.59–3.03)	< 0.001
81+	5.20 (4.80–5.60)	< 0.001	5.84 (5.39–6.31)	< 0.001
Racial		< 0.001		
API	Reference		Reference	
AIAN	1.37 (1.14–1.65)	< 0.001	1.31 (1.09–1.57)	< 0.001
Black	1.51 (1.41–1.62)	< 0.001	1.56 (1.46–1.67)	< 0.001
White	1.11 (1.05–1.17)	< 0.001	1.18 (1.12–1.25)	< 0.001
Surgery		< 0.001		
No	Reference		Reference	
Yes	0.62 (0.59–0.64)	< 0.001	0.72 (0.69–0.75)	< 0.001
Summary stage		< 0.001		
In situ	Reference		Reference	
Localized	2.5 (2.4–2.6)	< 0.001	1.27 (1.12–1.44)	< 0.001
Regional	5.1 (4.9–5.3)	< 0.001	1.36 (1.14–1.62)	< 0.001
Distant	15.9 (15.3–16.5)	< 0.001	3.03 (2.52–3.63)	< 0.001
AJCC stage		< 0.001		
Stage 0a	Reference		Reference	
Stage 0is	1.4 (1.3–1.5)	< 0.001	1.18 (1.10–1.26)	< 0.001
Stage I	1.9 (1.8–1.9)	< 0.001	1.39 (1.22–1.58)	< 0.001
Stage II	4.4 (4.3–4.6)	< 0.001	3.22 (2.83–3.66)	< 0.001
Stage III	4.7 (4.5–4.9)	< 0.001	3.38 (2.82–4.05)	< 0.001
Stage IV	10.9 (10.5–11.2)	< 0.001	5.48 (4.57–6.57)	< 0.001
Histologic type		< 0.001		
Transitional	Reference		Reference	
Adenocarcinomas	2.25 (2.05–2.46)	< 0.001	1.04 (0.95–1.15)	0.371
Epithelial	3.07 (2.89–3.27)	< 0.001	1.37 (1.29–1.46)	< 0.001
Squamous	3.32 (3.12–3.54)	< 0.001	1.77 (1.66–1.89)	< 0.001
Unspecified	2.82 (2.54–3.13)	< 0.001	1.20 (1.08–1.34)	< 0.001

*HR* hazard ratio, *CI* confidence interval, *AIAN* American Indian/Alaska Native, *API* Asian or Pacific Islander

The multivariate analysis results showed that the risk of bladder cancer was higher in patients who were male (HR = 0.99, 95% CI = 0.96–1.01, P = 0.336) than in patients who were female. Compared to patients aged 00–50 years, patients aged 51–60 years (HR = 1.34, 95% CI = 1.23–1.46, P < 0.001), 61–70 years (HR = 1.70, 95% CI = 1.57–1.85, P < 0.001), 71–80 years (HR = 2.80, 95% CI = 2.59–3.03, P < 0.001), and 81+ years (HR = 5.84, 95% CI = 5.39–6.31, P < 0.001) were significantly associated with increased mortality. When compared to the API group, the AIAN (HR = 1.31, 95% CI = 1.09–1.57, P < 0.001), black (HR = 1.56, 95% CI = 1.46–1.67, P < 0.001), and white (HR = 1.18, 95% CI = 1.12–1.25, P < 0.001) groups showed different survival probabilities. Compared to patients without surgical treatment, patients who underwent surgical treatment (HR = 0.72, 95% CI = 0.69–0.75, P < 0.001) had a higher survival rate. Compared to in situ grade tumors, localized grade (HR = 1.27, 95% CI = 1.12–1.44, P < 0.001), regional grade (HR = 1.36, 95% CI = 1.14–1.62, P < 0.001) and distant grade (HR = 3.03, 95% CI = 2.52–3.63, P < 0.001) tumors had increased HRs. As the tumor grade increased, the value of HR also increased. Compared to tumors in stage 0a, stage 0is (HR = 1.18, 95% CI = 1.10–1.26, P < 0.001), stage I (HR = 1.39, 95% CI = 1.22–1.58, P < 0.001), stage II (HR = 3.22, 95% CI = 2.83–3.66, P < 0.001), stage III (HR = 3.38, 95% CI = 2.82–4.05, P < 0.001), and stage IV (HR = 5.48, 95% CI = 4.57–6.57, P < 0.001) tumors had significantly increased HRs. Regarding different histologic types, the HR values of the types other than transitional cell carcinoma were higher, including adenocarcinoma (HR = 1.04, 95% CI = 0.95–1.15, P = 0.371), epithelial cell carcinoma (HR = 1.37, 95% CI = 1.29–1.46, P < 0.001), unspecified carcinoma (HR = 1.20, 95% CI = 1.08–1.34, P < 0.001), and squamous cell carcinoma (HR = 1.77, 95% CI = 1.66–1.89, P < 0.001), which had the highest mortality rate.

### Kaplan–Meier survival analysis

**Fig. 1:**
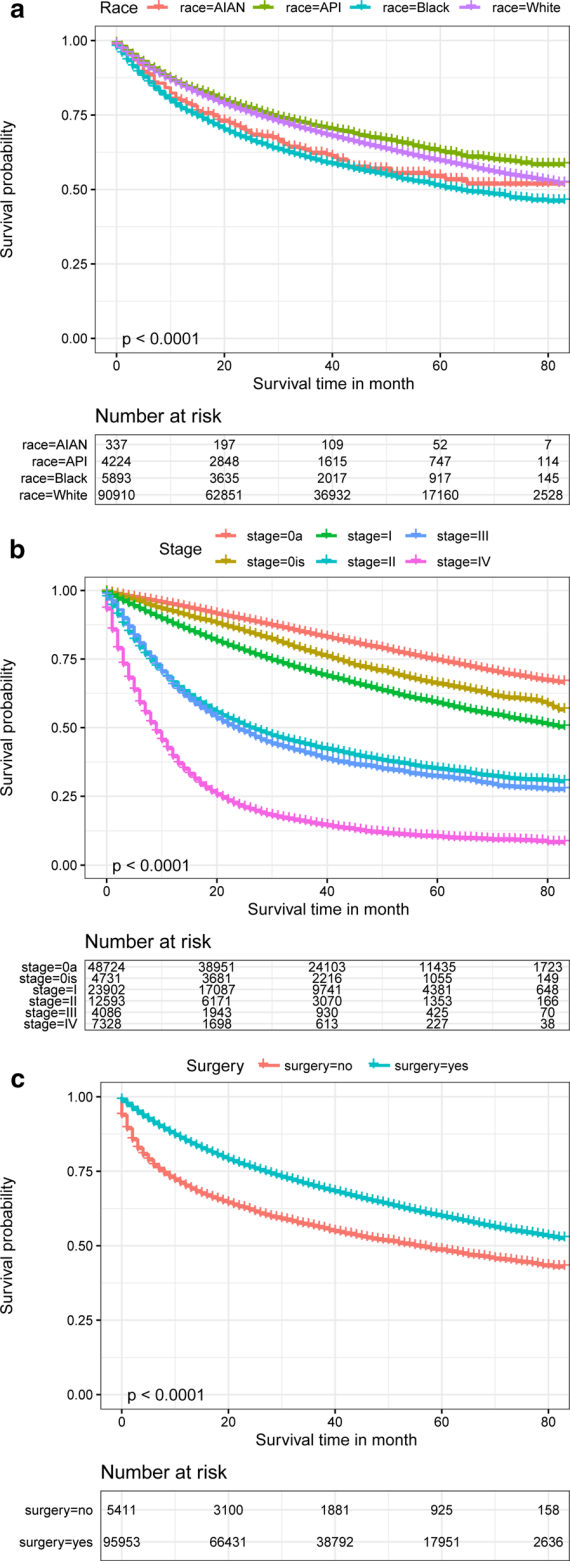
Kaplan–Meier survival curves for overall patients with bladder cancer in different conditions

**Fig. 2:**
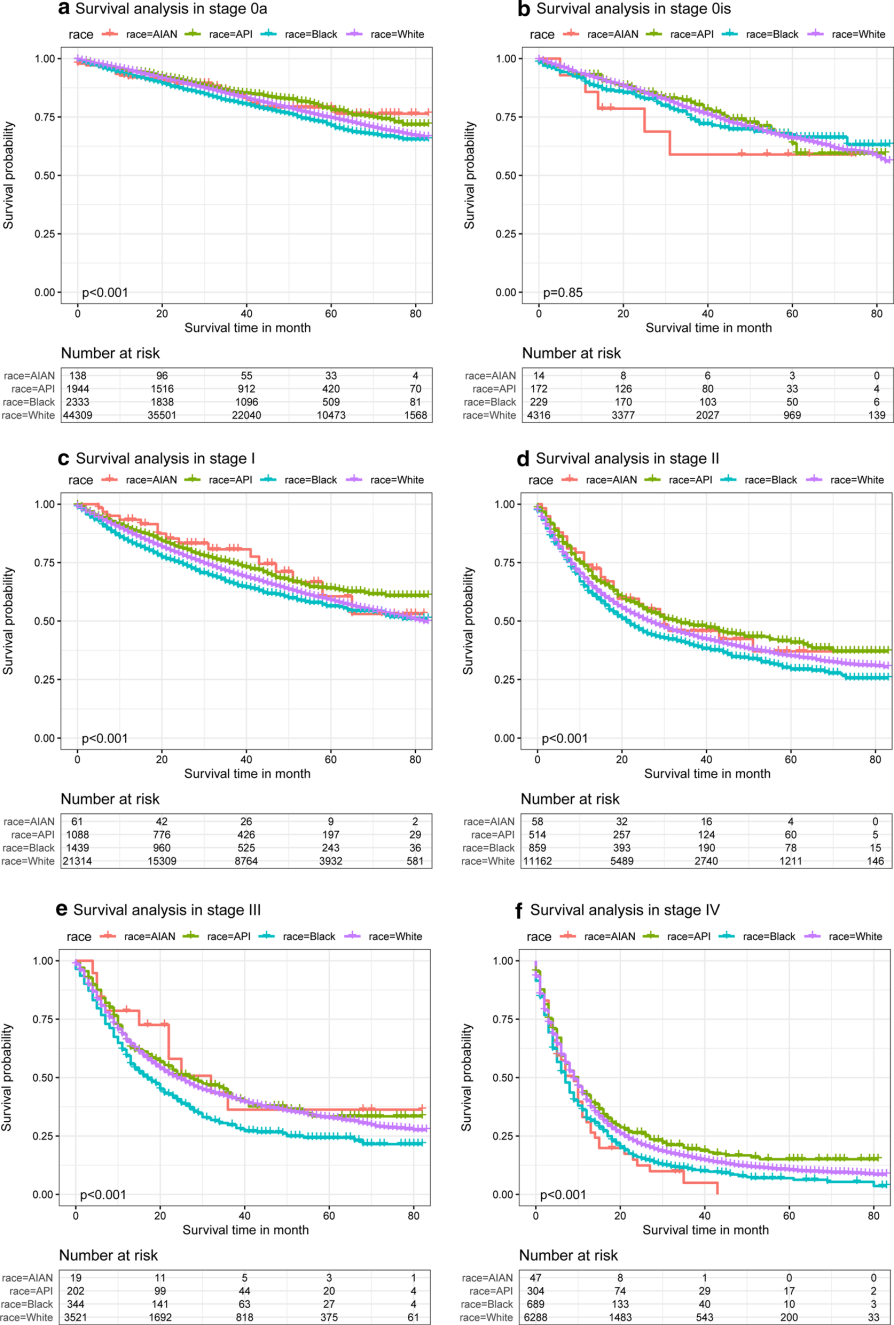
Kaplan–Meier survival curves for different stage in different race of bladder cancer patients. Kaplan–Meier survival curves in different grade of stage 0a (**a**), stage 0is (**b**), stage I (**c**), stage II (**d**), stage III (**e**), and stage IV (**f**)

**Fig. 3:**
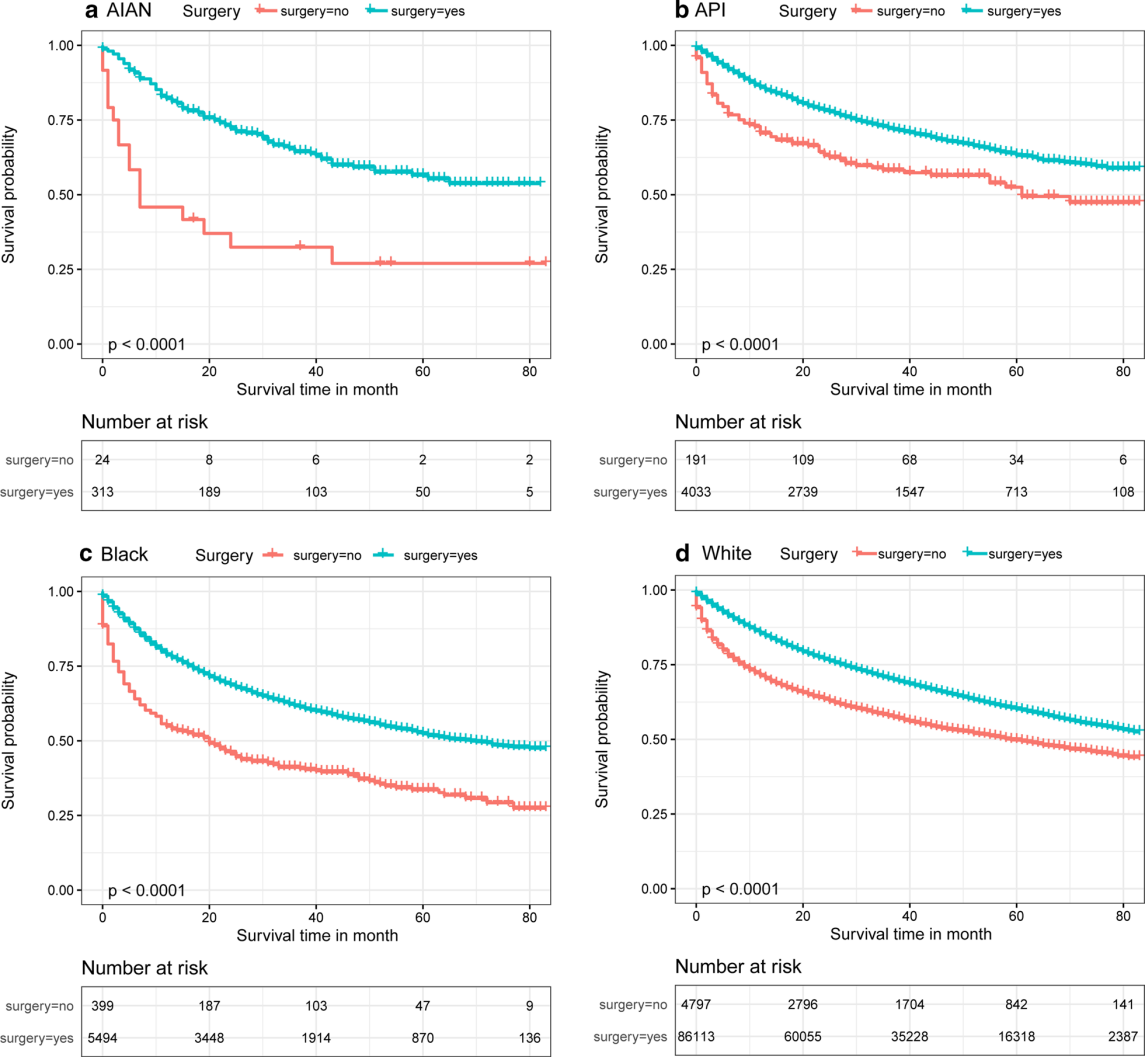
Kaplan–Meier survival for the difference of surgery and non-surgery in different race of bladder cancer patients

Figure 1a–c shows the survival curves for the overall patients of different subgroups. Figure 1a shows the survival curves for patients of different ethnicities. As shown in the figure, the survival probability of patients of each ethnicity except for black patients is > 50% at 80 months. The survival was highest for API patients, followed by white patients and AIAN patients, with the worst survival among black patients. As shown in Fig. 1b, there are significant differences in survival between patients in the early (stage 0a, stage 0is, stage I), middle (stage II, stage III) and late (stage IV) stages of disease. The survival probability for early-stage patients is > 50% at 80 months. In Fig. 1c, it is obvious that the survival probability of patients after surgery is much better than that without surgery. Figure 2a–f show the survival probabilities of different ethnicities at the six tumor stages (stage 0a, stage 0is, stage I, stage II, stage III, and stage IV). Figure 2a shows that the survival probability of these four ethnicities is very high. Even after 80 months, the survival of these patients was still greater than 62.5%. Overall, we can see that the four ethnicities were not significantly different in survival for the first few months of the 0a phase, but after approximately 40 months, the survival probability of white patients was highest, while that of black patients was lowest. For the data shown in Fig. 2b, the P value was 0.85, indicating that the results were not statistically significant. The main reason for this phenomenon is that there were very few patients in stage 0, particularly AIAN patients. For these small sample sizes, we had limited statistical power to detect differences between ethnicities. Stage I data are shown in Fig. 2c and show that during the first 50 months, AIAN patients had the highest survival probability, but for the remaining time, API patients had the highest survival probability. Figure 2d shows that the survival probability was consistently lowest for black patients, followed by white patients. API and AIAN patients showed nearly equal chances of survival. Figure 2e shows that most of the time, AIAN patients had the highest probability of survival. The survival probabilities of white and API patients were similar, and the survival probability of black patients was always the lowest. Figure 2f describes stage IV patients and shows that the order of survival from high to low was API, white, black, and AIAN patients. The AIAN patient data are particularly noteworthy, as these patients had a survival rate of 0 after 45 months of bladder cancer. Figure 3a–d shows the survival curves of patients of different ethnicities with and without surgery. As seen from the figure, the survival possibility of patients after surgical treatment was significantly improved. By comparing the distance between the two lines, we can judge the impact of surgery on the survival of patients. The greater the distance between the two lines is, the better the treatment effect of surgery on patients. Therefore, we know that the surgical treatment of AIAN and black patients is better than that of API and white patients.

### Nomogram

**Fig. 4:**
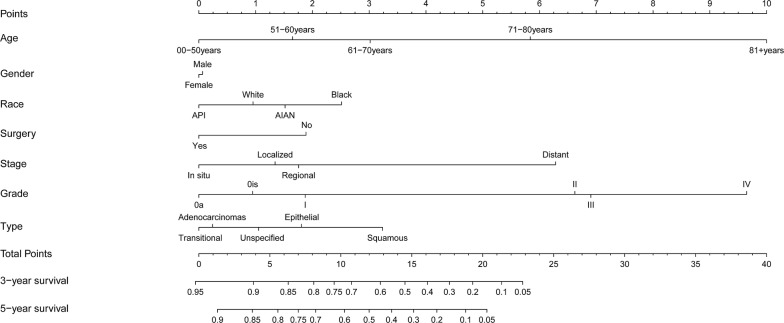
Nomogram of prediction for 3-year and 5-year overall survival of bladder cancer. Vertical line between each variable and points scale can be drawn to acquire points of each variable. Predicted survival rate was calculated according to the total points by drawing a vertical line from Total Points scale to overall survival scale

**Fig. 5:**
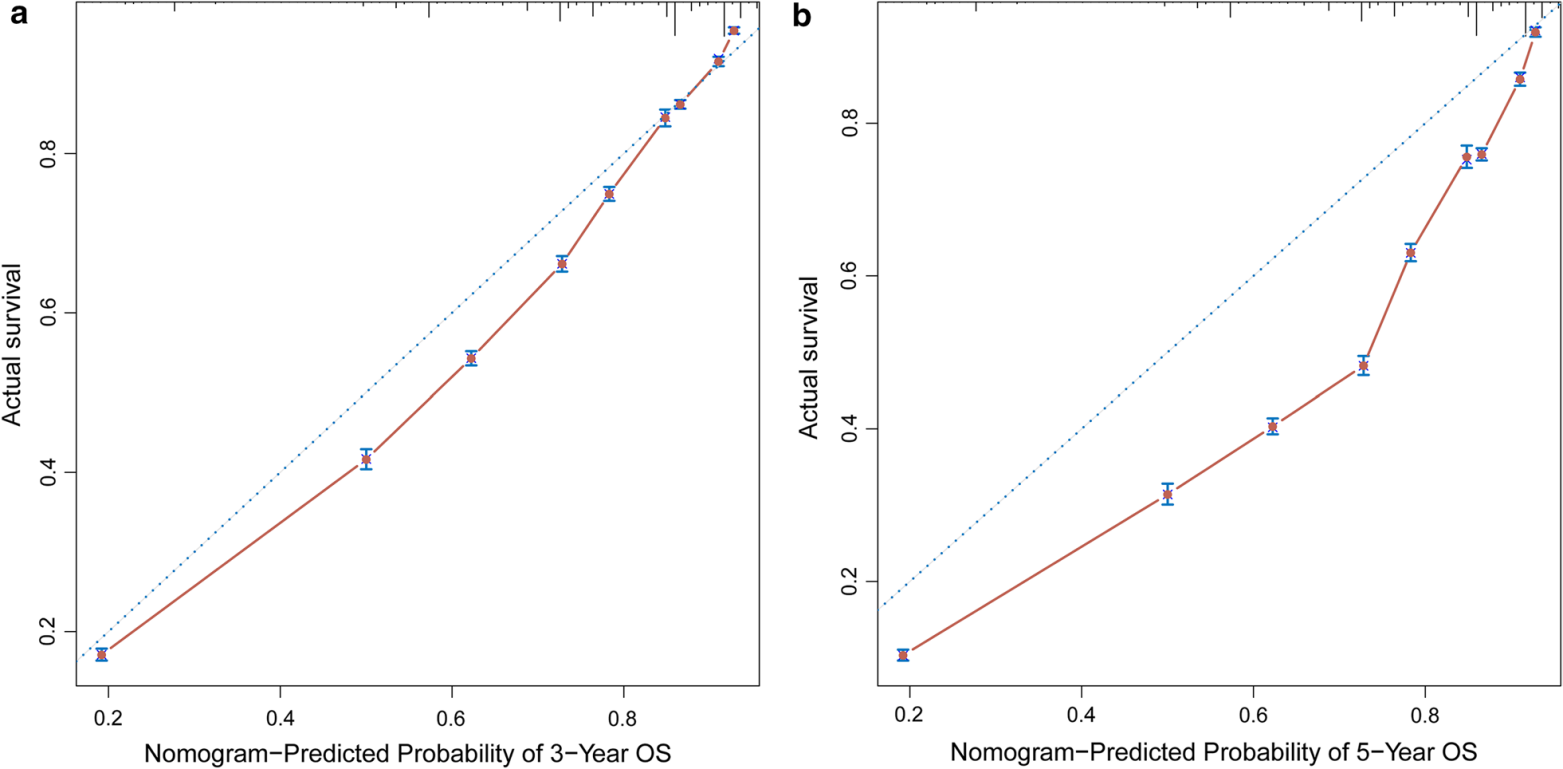
Calibration curves of the nomogram-predicted 3-year (**a**) and 5-year (**b**) overall survival

Based on the multivariate analysis results, we established a nomogram for OS (Fig. 4). To estimate the OS for 3 and 5 years, we first determined the total score for each person based on the sum of the dot scale at the top of the nomogram and the number of points for each factor. Finally, we estimated the OS rates for 3 and 5 years based on the fractional proportion at the bottom of the nomogram. The calibration plot based on bootstrap resampling validation demonstrated good agreement between the nomogram-predicted and observed survival rates (Fig. 5). The C-index was 0.782, suggesting that the nomogram was an accurate model for predicting OS.

## Discussion

Bladder cancer is the ninth most common cancer in the world [1] and one of the most common cancers of the urinary system [3]. Thus, we should pay more attention to bladder cancer. In this study, we obtained data from 101,364 bladder cancer patients. We established a nomogram to visually and effectively predict the 3- and 5-year OS of patients with bladder cancer. To our knowledge, previous studies have examined the incidence of bladder cancer and the treatment or inhibition of bladder cancer; however, whether there are racial differences in bladder cancer survival has not been explored. In this study, we found that there is a racial difference in the survival of bladder cancer patients, where black and AIAN patients with bladder cancer showed a lower survival probability than white and API patients.

The results of this study are similar to those of previous studies [23]: we found that independent of ethnicity, the number of male bladder cancer patients is far greater than that of female patients, and most patients are over 50 years old. Most bladder cancers are transitional epithelial cell carcinomas. Several studies have explained why bladder cancer is more likely to occur in males [16, 17, 24–27]; one reason is the interaction of estrogen, androgen, and the liver. The other reason is related to smoking because there are more male smokers than female smokers, and smoking increases the risk of bladder cancer. In addition, elderly patients are prone to bladder cancer due to the deterioration of the human immune system, which leads to an increase in the incidence of cancer. Moreover, with increasing age, detrusor activity becomes insufficient, leading to chronic urinary retention, which enhances exposure to carcinogens and increases the risk of developing disease [28–30].

Previous studies have shown that white individuals are more likely to develop bladder cancer than individuals of other ethnicities [23]. However, we cannot judge the difference in the incidence of bladder cancer among different ethnic groups from the results of this study. The results of this study only indicate the number of patients. We can only judge the incidence of cancer in different ethnicities using the standard incidence rate, as the proportion of ethnic groups and the total number of people in the US population is different, so these cannot be directly compared. However, the survival rate examined in this study is not affected by differences in the population distribution.

In this study, we used univariate and multivariate analyses. We used univariate analysis to determine whether each variable was meaningful to the study and then performed multivariate analysis on these factors. In general, patient survival is not controlled by a single factor. Therefore, to make our study results better reflect real life, we used multivariate analysis.

In agreement with that in previous studies, we did not find any significant differences in the risk of death between males and females. We found that the risk of death increased with increasing age and the level of cancer. Several studies have explained why patients’ survival possibility declines with age [31–33]. Overall, as patient age increases, immunity decreases, and patient physical function declines, which will affect their recovery. In addition, with increasing patient age, the overall exposure to carcinogens is likely to increase, which can affect patient survival. As the tumor grade increases, more tumor cells can spread, and treatment becomes more difficult. Consequently, the likelihood of patient survival will decrease.

Black and AIAN patients with bladder cancer showed a lower survival probability than patients of other races, and API patients showed the highest survival probability. While there is no research that definitively explains this phenomenon, some studies have described ethnic differences in chronic nephritis, myeloma and breast cancer [34–37]. Based on these results, we suspect that ethnic differences in the survival of patients with bladder cancer may be due to the combined effects of biology and epidemiology. There may be some genetic variations in different ethnicities that may affect the survival rate of bladder cancer patients by affecting the synthesis of proteins; moreover, the reasons for the different survival rates of bladder cancer patients may also be different ethnic habits and cultures. However, in addition to genes, ethnic habits and culture, there may be other reasons affecting the survival rate of bladder cancer that are worthy of future research.

From the results, we know that surgery can greatly improve the survival rate of patients. We also found some other interesting findings, and the results of the study showed that people of different ethnicities who underwent surgery had different outcomes. AIAN and black patients were able to benefit more from the surgery than the other two races. Surgery is generally performed to remove the lesion and can eliminate most cancer cells. There are also many factors affecting the prognosis of bladder cancer surgery, such as the type of bladder cancer in patients. The prognosis of noninvasive bladder cancer patients after surgery is better than that of invasive bladder cancer patients [38]. At the same time, the prognosis of early-stage patients is better than that of advanced-stage patients. The postoperative care of cancer patients also affects their prognosis. The better the postoperative care is, the better the patient prognosis. The use of adjuvant chemotherapy after resection also increases patient survival [39, 40].

This study is the first to create a nomogram for bladder cancer. Nomograms are widely used to predict patient survival [41, 42], and there are multiple factors that affect the survival of patients with bladder cancer. Based on multivariate Cox regression analysis, we identified age, sex, ethnicity, surgery, summary stage, AJCC stage, and histologic type as independent prognostic factors for OS. These data were then used to establish a nomogram to visually and effectively predict the 3-year and 5-year OS of patients with bladder cancer. The variables included in this nomogram were all independent factors influencing the survival of patients with bladder cancer and helped to better predict the survival of bladder cancer patients. Using this nomogram, we will be able to more accurately predict the survival of patients in the future. The C-index of the nomogram for this study was 0.782, indicating that the nomogram was an accurate model for predicting OS. The results of this study are particularly relevant for predicting patient survival.

This study also has certain limitations. First, the scope of the database was not large, as the SEER database only includes 27.8% of the US population. Due to the limited number of patients in some ethnicities, we lacked statistical power for analyzing survival differences in stage 0is patients. Consequently, our survival analysis results for this period were uninformative. The sample size of AIAN patients is relatively small, and it will have little impact when studying the differences between different ethnic groups with different tumor stages. Second, we did not examine other factors that could have affected the survival rate, such as economic conditions, type of surgical treatment, posttreatment state-of-care, and physical condition of the patient [43–45]. Adding these data would make the nomogram’s predictions more accurate, but these factors did not have a pronounced impact on our study results. Finally, both chemotherapy and radiation may affect the survival prognosis of patients. However, the original SEER data did not provide these factors. Therefore, these two factors may affect the stability of patients’ results.

## Conclusions

Our study showed that there are racial differences in the survival of patients with bladder cancer and that there is no significant difference in survival between black and AIAN patients, but the survival of these two races is worse than that of white and API patients; moreover, API patients have the best survival potential of the four ethnicities examined. The significant prognostic factors for OS in bladder cancer patients include age, sex, ethnicity, summary stage, AJCC stage, surgery type, and histologic type. AIAN and black patients were able to benefit more from surgery than the other two races.

## Data Availability

Not applicable.

## Abbreviations

AIAN: American Indian/Alaska Native
AJCC: American Joint Committee on Cancer
API: Asian or Pacific Islander
CI: Confidence interval
HR: Hazard ratios
OS: Overall survival
SEER: Surveillance, Epidemiology, and End Results
